# Nanoparticle uptake by airway phagocytes after fungal spore challenge in murine allergic asthma and chronic bronchitis

**DOI:** 10.1186/1471-2466-14-116

**Published:** 2014-07-16

**Authors:** Marianne Geiser, Christoph Wigge, Melanie L Conrad, Sylvie Eigeldinger-Berthou, Lisa Künzi, Holger Garn, Harald Renz, Marcus A Mall

**Affiliations:** 1 Institute of Anatomy, University of Bern, Bern, Switzerland; 2 Institute of Laboratory Medicine and Pathobiochemistry - Molecular Diagnostics, Philipps University of Marburg, Marburg, Germany; 3 Department of Psychosomatic Medicine, Charité-Universitätsmedizin Berlin, Berlin, Germany; 4 Department of Translational Pulmonology, Translational Lung Research Center, Heidelberg (TLRC), Member of the German Center for Lung Research (DZL), University of Heidelberg, Heidelberg, Germany

**Keywords:** Asthma, Clearance, COPD, Eosinophils, Fungal spores, Gold, Macrophages, Mouse, Nanoparticles, Phagocytosis

## Abstract

**Background:**

In healthy lungs, deposited micrometer-sized particles are efficiently phagocytosed by macrophages present on airway surfaces; however, uptake of nanoparticles (NP) by macrophages appears less effective and is largely unstudied in lung disease. Using mouse models of allergic asthma and chronic obstructive pulmonary disease (COPD), we investigated NP uptake after challenge with common biogenic ambient air microparticles.

**Methods:**

Bronchoalveolar lavage (BAL) cells from diseased mice (allergic asthma: ovalbumin [OVA] sensitized and COPD: Scnn1b-transgenic [Tg]) and their respective healthy controls were exposed *ex vivo* first to 3-μm fungal spores of *Calvatia excipuliformis* and then to 20-nm gold (Au) NP. Electron microscopic imaging was performed and NP uptake was assessed by quantitative morphometry.

**Results:**

Macrophages from diseased mice were significantly larger compared to controls in OVA-allergic versus sham controls and in Scnn1b-Tg versus wild type (WT) mice. The percentage of macrophages containing AuNP tended to be lower in Scnn1b-Tg than in WT mice. In all animal groups, fungal spores were localized in macrophage phagosomes, the membrane tightly surrounding the spore, whilst AuNP were found in vesicles largely exceeding NP size, co-localized in spore phagosomes and occasionally, in the cytoplasm. AuNP in vesicles were located close to the membrane. In BAL from OVA-allergic mice, 13.9 ± 8.3% of all eosinophils contained AuNP in vesicles exceeding NP size and close to the membrane.

**Conclusions:**

Overall, AuNP uptake by BAL macrophages occurred mainly by co-uptake together with other material, including micrometer-sized ambient air particles like fungal spores. The lower percentage of NP containing macrophages in BAL from Scnn1b-Tg mice points to a change in the macrophage population from a highly to a less phagocytic phenotype. This likely contributes to inefficient macrophage clearance of NP in lung disease. Finally, the AuNP containing eosinophils in OVA-allergic mice show that other inflammatory cells present on airway surfaces may substantially contribute to NP uptake.

## Background

Epidemiologists have consistently found an association between particulate air pollution and adverse health effects in humans [1,2]. Thereby, a specific toxicological role was attributed to ultrafine particles (UFP, particles with aerodynamic diameters ≤ 100 nm), and individuals with pre-existing respiratory and cardiovascular diseases were found to be more vulnerable to particle concentration increase in ambient air. Aerosol therapy in its modern form is an indispensable tool for topical treatment of pulmonary diseases and is increasingly used for systemic therapy [3]. The fast development of nanotechnology has brought about novel delivery strategies for inhaled and target-specific drugs for therapeutic applications, vaccines and diagnostics [4,5]. Gold nanoparticles (AuNP) are a candidate material for therapeutic applications, however little is known about how AuNP interact with different cell types [6]. Due to the high deposition efficiency of nanoparticles on the surface of the entire respiratory tract [7] and the health effects of UFP reported in humans (e.g. [8]), it is important to understand the interactions of deposited AuNP with target cells.

Macrophages, which patrol airway surfaces and are key players in lung defense, are one of the first cells to come into contact with inhaled and deposited particles. Surface macrophages function primarily to phagocytose deposited particles; clearing them from the airway surface and minimizing their impact on the epithelium and underlying tissues [9]. Phagocytosis of micrometer-sized particles is fast and essentially completed within 24 hours [10]. Uptake of NP by surface macrophages, however, seems to differ from that of micrometer-sized particles, as evidenced by inefficient phagocytosis of inhaled 20-nm titanium dioxide (TiO_2_) NP in the lungs of healthy rats [11]. Since particle uptake by macrophages is important for lung homeostasis, these cells also likely play a role in lung pathogenesis. Allergic asthma and chronic obstructive pulmonary disease (COPD) are the two most relevant inflammatory lung diseases in humans [12,13]. Allergic asthma is primarily an airway disease with goblet cell hyperplasia, increased airway reactivity and influx of eosinophils. The pathophysiology of COPD is caused by inhaled noxious particles and/or gases that trigger inflammatory responses in the conducting airways, i.e. chronic bronchitis, as well as a continuous destruction of the alveolar airspaces, i.e. emphysema. Symptomatically, patients suffer from chronic cough and dyspnea resulting from increased mucus secretion and airflow obstruction; impaired macrophage clearance of deposited particles is likely an important factor in the development and progression of both diseases.

In asthma, impaired macrophage clearance of micrometer-sized particles has been reported, e.g. bacteria in children [14] and 2-μm polystyrene microspheres in murine experimental asthma [15]. Submicrometer particles, however, were more rapidly phagocytosed by sputum macrophages of asthmatics than healthy subjects [16]. In COPD, phagocytic activity of surface macrophages seems to be decreased independent of particle size; e.g. reduced uptake of apoptotic cells has been reported [17], and we recently obtained evidence for delayed and less efficient uptake of inhaled 20-nm AuNP in Scnn1b-Tg mice with COPD-like lung disease [18]. Contrary to the consequences of harmful NP, impaired phagocytosis resulting in a prolonged residence time of deposited AuNP on the epithelial surface may be favorable in case of therapeutic targeting of the lung parenchyma by inhaled aerosols.

Fungal spores are ubiquitous components of occupational and residential indoor as well as outdoor air, and they have long been associated with allergic and non-allergic respiratory diseases [19-21]. The quantities of airborne fungal spores are generally 100 - 1000 times greater than those of pollen grains; there are usually 10^3^ - 10^4^ spores/m^3^ air with peak values of 10^6^ spores/m^3^[22]. Contrary to pollen grains, the regional differences in the outdoor fungal spore occurrence are rather small, at least in the northern hemisphere. *Basidiomycetes* represents the largest and most complex group of fungi, and their spores regularly constitute the major portion of the spore-load in ambient air [23,24]. Our respiratory tract is frequently challenged by inhaled basidiospores, e.g. from *Calvatia* species, which are 3 - 5 μm in diameter and are known to deposit in conducting airways and alveoli [25,26]. In order to unravel the mechanisms of beneficial or adverse effects of inhaled nanoparticles, the role of other, frequently inhaled particles with known participation in lung disease need to be included in model studies. Knowledge regarding NP uptake by surface macrophages after a challenge with common air constituents such as fungal spores is lacking in the literature.

This study aimed to investigate the uptake and localization of AuNP at the ultrastructural, i.e. individual particle level, in spore-challenged bronchoalveolar (BAL) cells harvested from murine models of common inflammatory lung diseases, (i) in OVA-induced experimental allergic asthma, which is characterized by acute lung inflammation with increased BAL eosinophils [27], and (ii) in COPD, represented by Scnn1b-Tg mice, which mimic key aspects of chronic obstructive lung diseases in humans, including chronic bronchitis with airway mucus obstruction, inflammation with increased BAL leukocytes and structural lung damage [28-30].

Combining exposure to a potential inhalative nanocarrier with ultrastructural analysis of macrophages in healthy and diseased lungs, we report here the mode and efficiency of macrophage AuNP clearance in the presence of ubiquitous biogenic ambient air microparticles. We further demonstrate effects of (i) disease status, (ii) airway surface leukocyte populations and (iii) mouse strain on macrophage AuNP clearance, which are all relevant for their use in therapeutic applications.

## Methods

### Experimental models of airway disease

**Figure 1:**
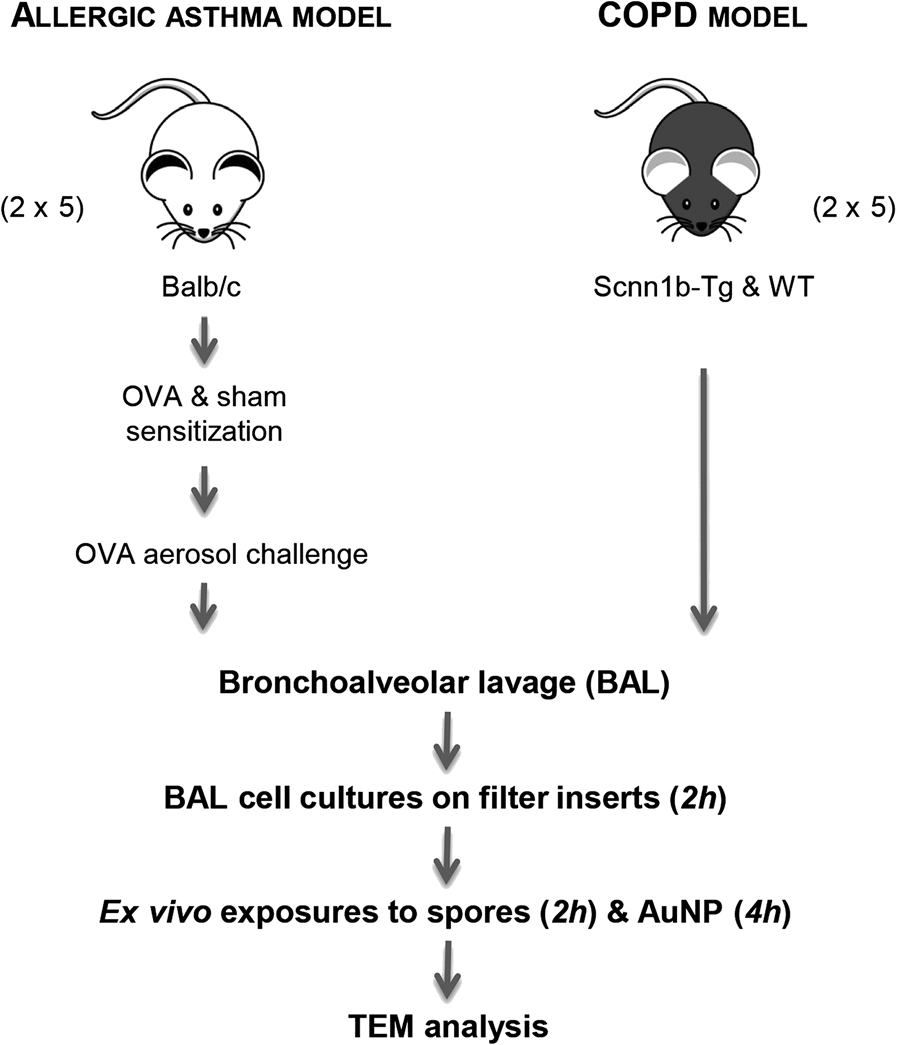
**Illustration visualizing the study design.** BAL cells of two well-described mouse models of lung diseases (OVA-allergic and Scnn1b-Tg, and their respective controls, i.e. sham-sensitized and WT mice) were sequentially exposed *ex vivo* to fungal spores and AuNP to assess their particle uptake capability by TEM and morphometric measurements.

Murine models were used to investigate particle uptake in conditions representing allergic asthma and COPD as well as in corresponding healthy controls (Figure 1). Recovery, treatment and analysis of BAL cells were identical in all animal groups.

Allergic airway inflammation was induced using an adjuvant-free experimental asthma protocol as previously described [27]. Briefly, animals (ten female BALB/c mice aged 6 - 8 weeks purchased from Harlan Winkelmann, Borchen, Germany) were sensitized by subcutaneous injection of 10 μg OVA (grade VI, Sigma, Steinheim, Germany, containing 1 ng of endotoxin per 10 μg OVA [dose used for sensitization]) in 200 μL phosphate buffered saline (PBS, Sigma, Steinheim, Germany) or sham injections of PBS on days 0, 7 and 14. Allergic airway inflammation was induced by 20 minutes of 1% OVA or sham PBS aerosol exposure on days 26, 27 and 28. BAL cell isolation was performed 24 h after the last allergen challenge.

The Scnn1b-Tg mouse is an established model of chronic inflammatory lung disease exhibiting key features of COPD and CF such as chronic airway inflammation, mucus obstruction and structural lung damage [28-32]. Five Scnn1b-Tg mice (airway targeted overexpression of the epithelial Na^+^ channel β subunit Scnn1b; line 6608 generated and maintained on a mixed C3H/HeN:C57BL/6N genetic background as previously described [30] and five WT littermates aged 16 - 18 weeks, were bred and reared at the University of Heidelberg, Heidelberg, Germany. Mouse progeny were genotyped by tail biopsy polymerase chain reaction (PCR) as previously described [33]. Animals were housed under a 12 h light/dark cycle, controlled humidity of 55% and temperature of 22°C, with access to food and water *ad libitum*.

For cell retrieval, animals were deeply anesthetized by intraperitoneal injection of 300 mg/Kg ketamine hydrochloride plus 30 mg/Kg xylazine (Rompun, Bayer Health Care, Leverkusen, Germany) prior to exsanguination by cutting the abdominal aorta and lung lavage.

All experiments were performed under federal guidelines for the use and care of laboratory animals and were approved by the responsible local authorities (Regierungspräsidium Giessen, Germany; Regierungspräsidium Karlsruhe, Germany; and the Cantonal Veterinary Office, Bern, Switzerland) and included ethical evaluation.

### Particles

Basidiospores of the *Calvatia* species were selected, since these spores have been well characterized in terms of their morphology and surface properties as well as their distribution, retention and clearance by macrophages in rodent lungs upon inhalation [26,34]. In addition, all analyses were performed in comparison to other particle types of the same size [9,34]. Fungal spores of *Calvatia excipuliformis* (3.5 μm in diameter), collected from dried mushrooms as previously described [26], were suspended at a density of 2 × 10^7^ particles/mL in fluorocarbon FC-72 (3 M, Neuss, Germany).

Similarly, valuable background information on AuNP for this in-vitro study was retrieved from very recent inhalation experiments with spark generated 20-nm AuNP using the same COPD animal model [18]. Colloidal gold particles (AuNP, 20 nm in diameter), suspended at 1 × 10^11^ particles/mL in citrate buffer, were obtained from Plano (Wetzlar, Germany).

### BAL cell isolation, cell culture and particle exposure

Lungs were lavaged with 5 × 1 mL sterile PBS as described previously [11]. Total number of BAL cells was determined using a Neubauer haemocytometer chamber. Differential cell counts (200 BAL cells per animal) were performed either on cytospin preparations (OVA-allergic and sham-sensitized mice) fixed and stained with Diff-Quick (Merz & Dade AG, Düdingen, Switzerland) or directly on filter-insert cell preparations (Scnn1b-Tg and WT mice) fixed with phosphate buffered 2.5% glutaraldehyde (EMS, Fort Washington, PA, USA). Macrophages, lymphocytes, eosinophils and neutrophils were differentiated by standard morphologic criteria.

For each animal, 3 × 10^5^ BAL cells in 100 μL complete medium (Dulbecco’s Modified Eagle’s Medium, D-MEM, supplemented with 10% fetal calf serum, FCS) were seeded on a micro-porous filter insert (pore size: 0.4 μm, growth area: 0.3 cm^2^, i.e. 1 × 10^6^ cells/cm^2^; Falcon, Becton Dickinson, Franklin Lakes, NJ, USA) in 24-well plates (Falcon), with each well containing 800 μL complete D-MEM. Cells were then cultured for 2 h at 37°C, 5% CO_2_ and 90% RH to select for adherent cells, i.e. macrophages and activated leukocytes (eosinophils, neutrophils, lymphocytes) that were freshly recruited to the lung surface. Non-adherent cells were washed off with serum-free D-MEM. Thereafter, sequential exposure of cells to the particles followed. Like this, concurrent exposure of micro- and nanoparticles was avoided to omit passive carriage of the smaller (nano)particles on the surface of the larger (micrometer sized) ones. To challenge phagocytosis, the remaining cells were first exposed to 1 × 10^6^ fungal spores (~ 3 spores/cell) in 100 μL serum-free D-MEM/insert for 2 h. Subsequently, non-adherent spores were washed off with 300 μL serum-free D-MEM/insert and BAL cells were then exposed to 1 × 10^9^ AuNP (~ 3 × 10^3^ NP/cell) in 100 μL serum-free D-MEM (zeta potential ~ 22 mV [35]) for 4 h. Non-adherent AuNP were rigorously washed off with 2 × 300 μL serum-free D-MEM/insert and 1 × 500 μL PBS. BAL cells were then successively fixed with 500 μL of 2.5% glutaraldehyde (EMS) in 0.03 M potassium-phosphate buffer, 1% osmium tetroxide in 0.1 M sodium-cacodylate buffer and 0.5% uranyl acetate in 0.05 M maleate buffer (Sigma-Aldrich, Buchs, Switzerland), dehydrated in a graded series of ethanol and embedded in epon resin (Sigma-Aldrich). Ultrathin sections of 50 nm nominal thickness were cut perpendicular to the filter-insert with an ultra-microtome (Ultratome, LKB, Stockholm, Sweden), mounted onto formvar-coated 200-mesh copper grids and post-stained with uranyl acetate and lead citrate (Ultrastain, Leica, Glattbrugg, Switzerland). For electron tomography, 150-nm sections were cut, mounted and stained as described above.

### Microscopic analysis of cells and particles

BAL cell morphology was first evaluated on toluidine blue stained sections of 1 - 2 μm nominal thickness using a Leica light microscope (Leica, Wetzlar, Germany). Ultrastructural analysis of cells and particles was performed on random ultrathin sections of the cells on filter inserts using a CM12 transmission electron microscope (TEM, Phillips, Eindhoven, The Netherlands) operated at 80 kV. For each animal model, approximately 150 cells were analyzed by TEM. Fungal spores and AuNP were unambiguously identified according to their size, shape and contrast. The diameters of macrophages, vesicles and particles are reported, since this is the common measure in cell biology. Equivalent diameters were calculated from section profile area measurements of the cell, vesicle or particle using the iTEM software (Olympus Soft Imaging Solutions, Münster, Germany).

For electron tomography, the 3D configuration of AuNP and surrounding structures was acquired by projection reconstruction of a multi-angular tilt series of thick sections (~ 150 nm) [36]. Electron tomography was performed on five vesicles containing AuNP using a Tecnai F20 field emission TEM (FEI Company, Eindhoven, Netherlands) operating at 200 kV. Typical tilt series were acquired at a magnification of 40,000× covering an angular range from -66° to 66° (with an increment of 2°) and a defocus of 0.53 μm. The pixel size in the tomogram was 0.51 nm. Tilt images were aligned semi-manually using the FEI software package (Inspect 3D, Amira, FEI Company, Eindhoven Netherlands). Three dimensional reconstructions of AuNP and segmentation of macrophage vesicles in the tomogram were performed manually with the segmentation editor implemented in Amira software.

### Statistics

BAL cells from all animal groups were treated the same way, i.e. first with spores and then with AuNP. Thus particle treatment was a within-subject (single measure) factor, while animal model (mouse strain) as well as allergy and COPD status was a between-animal factor. Due to the applied sampling design of analyzing all AuNP-containing vesicles in a macrophage, the number of data points obtained varied between macrophages and animals. Data of BAL cell number, macrophage size and distance of particles to vesicle membrane were analyzed by the non-parametric Mann-Whitney Rank Sum Test for two group comparisons and one-way analysis of variance (ANOVA) followed by Dunn or Holm-Sidak post hoc tests for pairwise multiple group comparisons. These analyses were performed using SigmaPlot 12 software (Systat Software Inc., San Jose, CA, USA). Data of vesicle to particle size ratios, R_V/P_ were analyzed for differences in the mechanism of AuNP uptake. A linear mixed effects model with a random intercept for each animal and fixed effects for health status, disease model/mouse strain and their interaction health status*disease model and vesicle class was used to model the logarithm of R_V/P_ (log[R_V/P_]), where the log transformation was chosen for linearization and diagnostic reasons. These analyses were performed with R software version R-2.13.0. A P-value < 0.05 was considered to be statistically significant. Descriptive statistics such as number of observations, mean, standard error of the mean (SEM) and range (minimum, maximum) are used to summarize the continuous outcome variables.

## Results

### Morphology of fungal spores and AuNP

**Figure 2:**
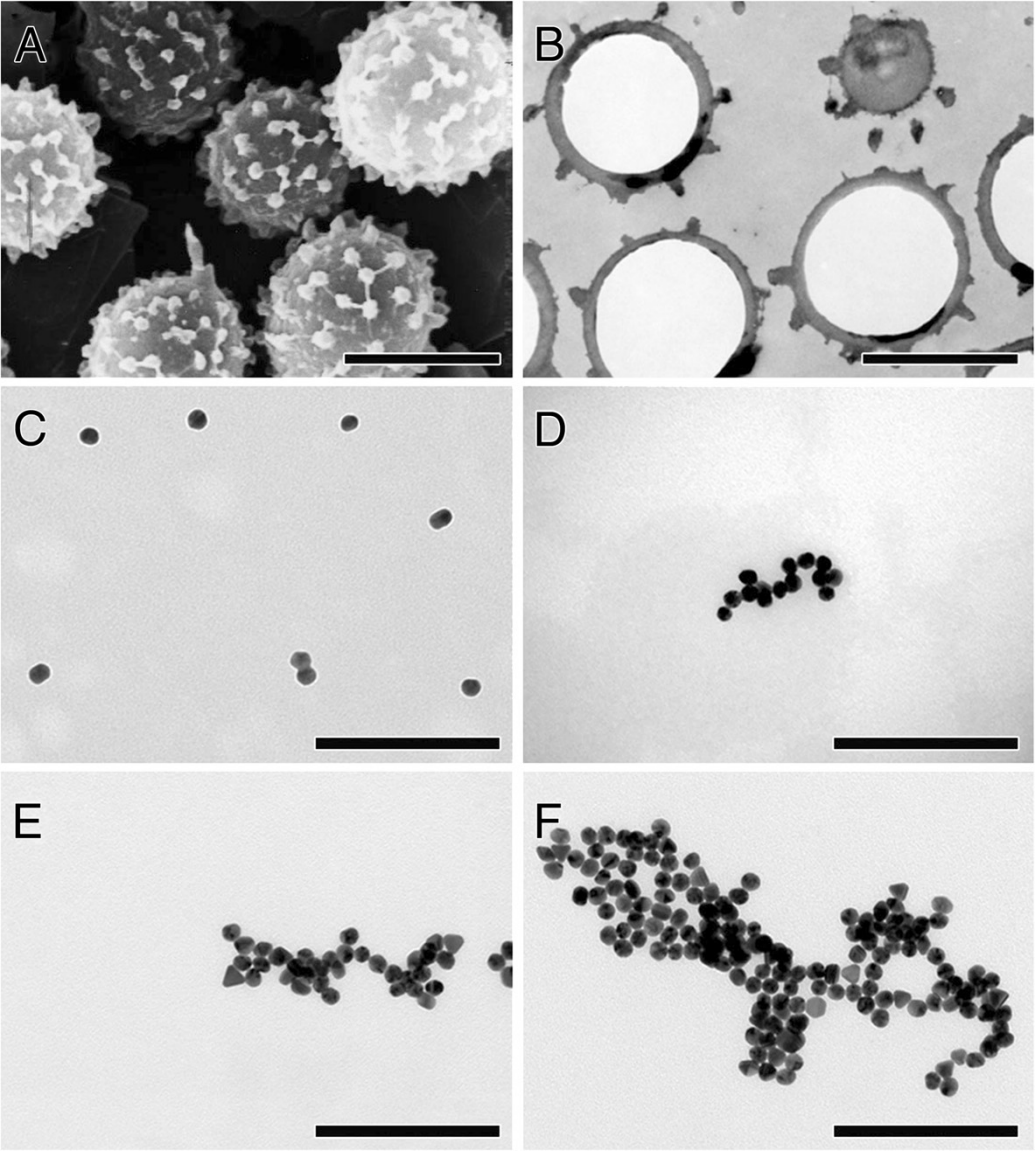
**Electron micrographs of micro- and nanoparticles. (A)** Scanning electron micrograph of sphere-shaped fungal spores with spikes. **(B)** Transmission electron micrograph of ultrathin section of epon-embedded spores. **(C-F)** Transmission electron micrographs of AuNP in serum-free D-MEM: **(C)** single NP and small cluster of two NP, **(D-F)** NP clusters of various sizes. Bars: **(A, B)** = 3 μm; **(C-F)** = 200 nm.

As shown in Figure 2, fungal spores are sphere-shaped with spikes and a geometric diameter of 3.5 ± 0.2 μm (n = 120) [26]. Single AuNP are spheres with a geometric diameter of 20.8 ± 3.4 nm (n = 100). AuNP in stock solution were mostly single particles, whereas we found agglomerates of different sizes, when AuNP were suspended in serum-free cell culture medium.

### BAL cell number, differential cell counts and cell morphology

As shown in Table 1, the mean number of total BAL cells obtained from OVA-allergic mice (n = 5) was significantly higher (P = 0.001) compared to healthy controls (n = 5). BAL cells from allergic mice consisted of 56.6 ± 3.8% eosinophils, 25.9 ± 2.4% macrophages, 10.4 ± 0.5% neutrophils and 9.1 ± 1.6% lymphocytes. In sham controls, 99.6 ± 0.4% of BAL cells were macrophages. The mean number of total BAL cells from Scnn1b-Tg animals (n = 5) was not significantly different from WT mice (n = 5). BAL cells from Scnn1b-Tg mice consisted of 97.8 ± 0.3% macrophages and 2.2 ± 0.3% lymphocytes (Table 1). In WT animals, 99.8 ± 0.2% of the cells were macrophages.

**Table 1:** Mean number and differential counts of BAL cells

**Animals**	**BAL**_**total**_ **× 10**^**6**^	**% Mac**	**% PMN**	**% Eos**	**% Lymph**
**OVA-allergic**	5.3^*^ ± 0.5	25.9^*^ ± 2.4	10.4^*^ ± 0.5	56.6^*^ ± 3.8	9.1^*^ ± 1.6
**Sham**	0.8 ± 0.3	99.6 ± 0.4	0	0	0
**Scnn1b-Tg**	0.6 ± 0.1	97.8 ± 0.3	0	0	2.2^*^ ± 0.3
**WT**	0.7 ± 0.1	99.8 ± 0.2	0	0	0

Mean values of n = 5 animals per group ± SEM. ^*^Significant difference to control group (P < 0.05). Mac, macrophages; PMN, polymorphnuclear leukocytes (neutrophils); Eos, eosinophils; Lymph, lymphocytes.

**Figure 3:**
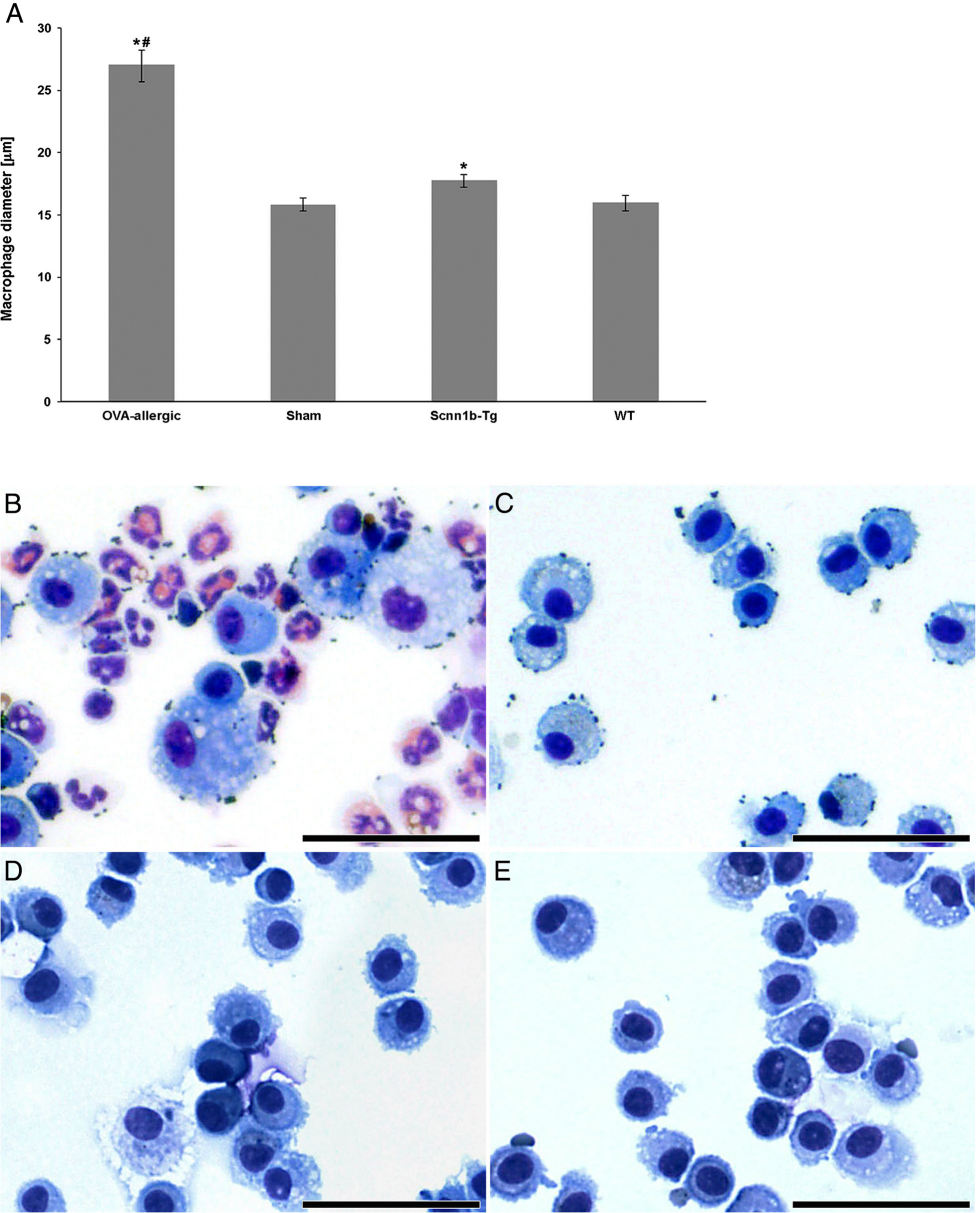
**Size distribution and morphology of macrophages. (A)** Size distribution of macrophages: Data are expressed as mean values ± SEM per animal group; ^*^significant difference to corresponding healthy controls (P < 0.05); ^#^significant difference to all other animal groups (P < 0.001). **(B-E)** Light micrographs of BAL cells: from **(B)** OVA-allergic and **(C)** sham-sensitized, **(D)** Scnn1b-Tg and **(E)** WT mice. Bars: 50 μm.

As shown in Figure 3, BAL macrophages from OVA-allergic and sham-sensitized mice appeared structurally normal, although, those of OVA-allergic animals were significantly larger (27.0 ± 1.3 μm in diameter, range 7.1 - 46.3 μm) compared to sham controls (15.8 ± 0.5 μm, range 8.2 - 25.2 μm) and any other animal group analyzed (P < 0.001). The macrophage population of Scnn1b-Tg mice appeared morphologically heterogeneous; some cells were abnormally large and many had a “foamy” cytoplasm as previously demonstrated [30,32]. WT macrophages showed normal morphology. As Figure 3 also demonstrates, Scnn1b-Tg macrophages were significantly larger (17.7 ± 0.5 μm diameter, range 5.3 - 36.2 μm) than those of WT mice (15.9 ± 0.6 μm, range 7.5 - 26.7 μm) (P = 0.014).

### Uptake and localization of particles

**Figure 4:**
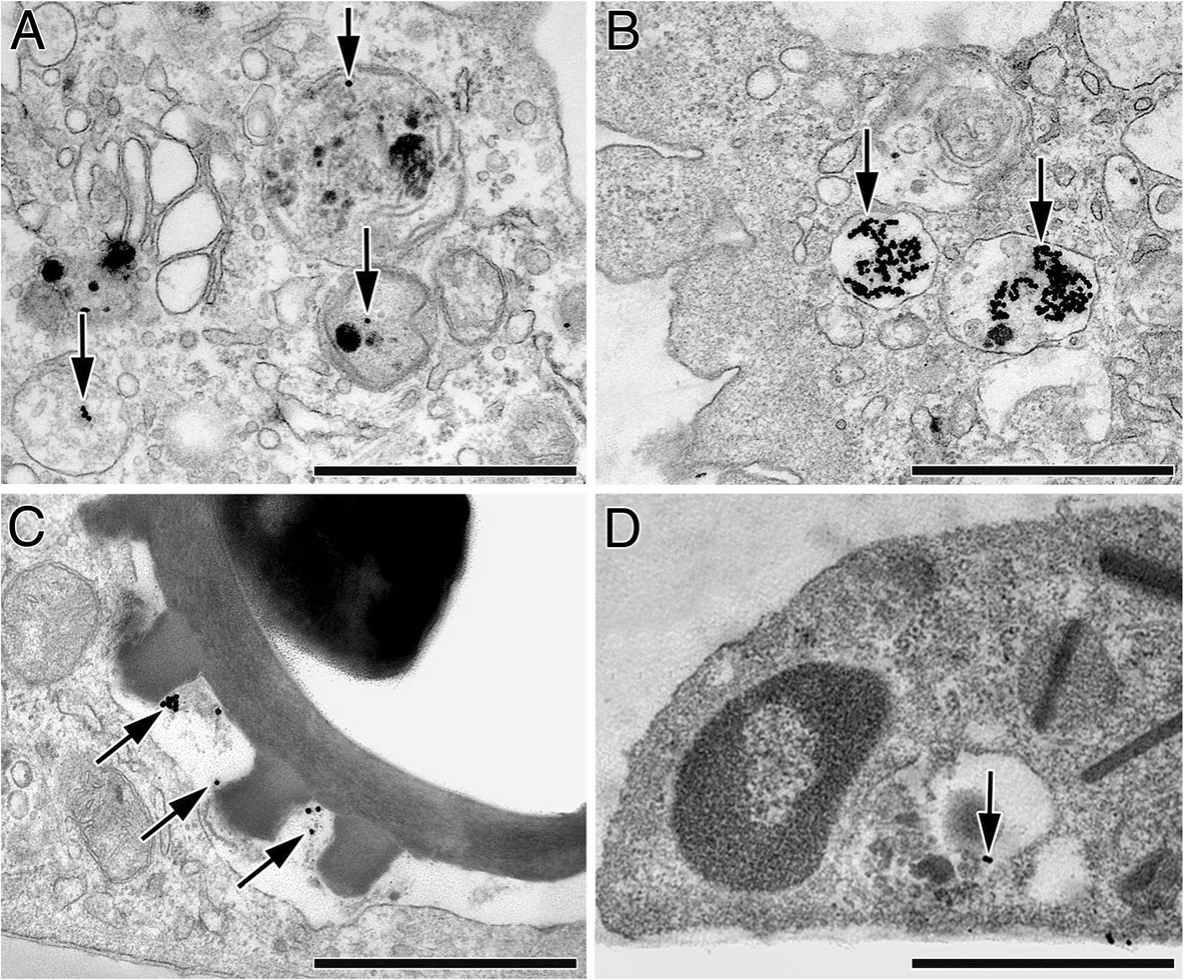
**Transmission electron micrographs of internalized AuNP (arrows) and spores in BAL cells. (A-C)** AuNP in macrophages: **(A)** Single AuNP and small cluster in vesicles largely exceeding NP size and containing substances of mostly unknown origin. **(B)** Large AuNP clusters in vesicles slightly larger than the NP cluster. **(C)** AuNP co-localized with fungal spore in phagosome. **(D)** Small AuNP cluster in an eosinophil of an OVA-allergic mouse, in a vesicle exceeding NP size and containing other electron dense material of unknown origin. Bars: 1 μm.

Electron microscopic and morphometric analysis of AuNP uptake by BAL cells revealed that a similar percentage of macrophages from OVA-allergic animals and sham controls contained AuNP (61.0 ± 21.4% vs. 62.1 ± 5.7% respectively). The percentage of BAL macrophages from Scnn1b-Tg mice containing AuNP tended to be lower (74.9 ± 7.2%) than in WT animals (82.0 ± 3.3%), though not statistically significant with the number of animals included in this study. Comparison of AuNP uptake by macrophages obtained from healthy control groups revealed significantly higher percentages of macrophages containing AuNP in naïve WT, i.e. C3H/HeN:C57BL/6N mice compared to sham-sensitized BALB/c mice (P = 0.016).In all animal groups, AuNP were found either as single particles in vesicles largely exceeding their size and containing other electron dense material of mostly unknown origin (Figure 4A), as agglomerates in vesicles of similar size (Figure 4B) or co-localized with spores in phagosomes (Figure 4C).

**Figure 5:**
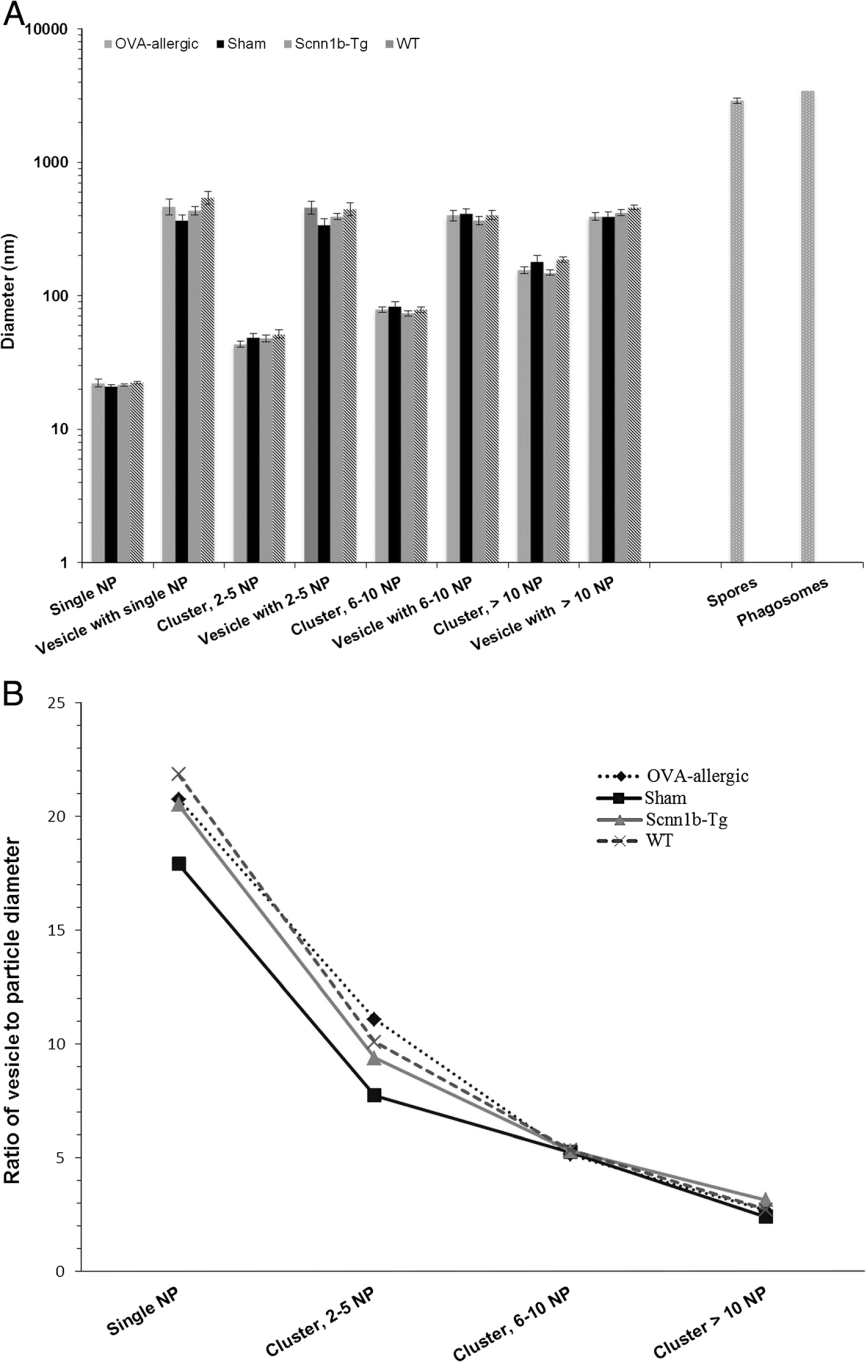
**Size distributions of vesicles and internalized particles in BAL macrophages. (A)** Sizes of AuNP and vesicles containing the particles. Data are expressed as mean values ± SEM per animal group. **(B)** Ratio of vesicle to particle diameter (R_V/P_).

As shown in Table 2 and Figure 5, the sizes of AuNP clusters in macrophage vesicles increased according to the number of agglomerated particles, while the sizes of the vesicles enclosing the AuNP remained constant, irrespective of AuNP cluster size in all experimental groups (Figure 5A). Thus, the ratio of vesicle to particle diameter R_V/P_ (Figure 5B) was large, i.e. ~ 20 for single AuNP and ~ 10 for small clusters of 2 - 5 AuNP; it then decreased to ~ 5 when clusters contained 6 - 10 AuNP and approached R_V/P_ ~ 1, i.e. the value representing phagocytic particle uptake, when clusters consisted of more than 10 AuNP. The supplementary statistical analysis of R_V/P_ data (Table 3) revealed no significant difference in AuNP uptake between macrophages from (i) the healthy and diseased animals, (ii) the two mouse strains or (iii) the diseased animals of the two groups. However, a significant vesicle class effect is found. The three coefficients given in Table 3 correspond to the effect of not being in vesicle class A (single AuNP) but in the respective category B (2 - 5 AuNP), C (6 -10 AuNP) or D (> 10 AuNP). All these three differences to vesicle class A are significant and the predicted log(R_V/P_), thus also the R_V/P_ itself, decreases with increasing number of nanoparticles.

**Table 2:** Sizes of vesicles and internalized particles

**Animals**	**Single NP**	**Vesicle**	**R**_**V/P**_	**Cluster**	**Vesicle**	**R**_**V/P**_	**Cluster**	**Vesicle**	**R**_**V/P**_	**Cluster**	**Vesicle**	**R**_**V/P**_
		_**1 NP**_		_**2-5 NP**_	_**2-5 NP**_		_**6-10 NP**_	_**6-10 NP**_		_**> 10 NP**_	_**> 10 NP**_	
**OVA-allergic**	22.2 ± 1.4	466 ± 64	20.8 ± 2.5	43.4 ± 2.3	459 ± 53	11.1 ± 1.2	78.7 ± 3.3	401 ± 38	5.1 ± 0.4	156 ± 9	393 ± 25	2.7 ± 0.2
	(n = 16)			(n = 26)			(n = 22)			(n = 38)		
**Sham**	21.0 ± 0.7	367 ± 38	17.9 ± 1.9	48.6 ± 3.3	338 ± 41	7.7 ± 1.3	83.5 ± 6.5	414 ± 34	5.2 ± 0.5	182 ± 19	393 ± 32	2.4 ± 0.2
	(n = 22)			(n = 28)			(n = 22)			(n = 30)		
**Scnn1b-Tg**	21.5 ± 0.3	435 ± 30	20.5 ± 1.4	47.9 ± 2.5	393 ± 22	9.4 ± 0.6	74.0 ± 3.3	366 ± 25	5.3 ± 0.4	149 ± 7^*^	420 ± 19	3.1 ± 0.2
	(n = 69)			(n = 87)			(n = 49)			(n = 77)		
**WT**	22.5 ± 0.4	543 ± 61	24.5 ± 2.8	50.4 ± 3.3	451 ± 43	10.3 ± 1.1	79.1 ± 3.7	404 ± 33	5.3 ± 0.5	188 ± 9	460 ± 20	2.7 ± 0.2
	(n = 46)			(n = 57)			(n = 34)			(n = 74)		

Data are presented as mean values ± SEM. ^*^Significant difference to control group (P = 0.005); n = number of measurements (vesicle and enclosed AuNP). R_V/P_ = ratio of vesicle to particle diameter.

**Table 3:** **Dependence of R**_**V/P **_**on health status, disease model/mouse strain and AuNP uptake**

**Animal group/Vesicle class**	**Fixed effect estimates**			
	**Value**	**SE**	**DF**	**P-value**
**(Intercept )**	2.941	0.103	677	0.000
**Healthy (Sham, WT)**	-0.137	0.129	13	0.307
**COPD model (Scnn1b-Tg, WT)**	-0.044	0.118	13	0.717
**Healthy, COPD model (WT)**	0.172	0.162	13	0.309
**Vesicle class B (2 - 5 NP)**	-0.817	0.056	677	0.000^*^
**Vesicle class C (6 - 10 NP)**	-1.351	0.063	677	0.000^*^
**Vesicle class D (> 10 NP)**	-2.004	0.055	677	0.000^*^

The estimates of the linear mixed effects model revealed no significant difference between (i) the healthy and diseased animals, (ii) the disease model, the two mouse strains or (iii) the diseased animals of the two groups on the log of the ratio of vesicle to particle diameter, log(R_V/P_). ^*^Significant vesicle class effect (P < 0.05), i.e. effect of not being in vesicle class A but in the respective category B, C or D; thus R_V/P_ decreases with increasing number of nanoparticles. SE = standard error; DF = degrees of freedom.

**Figure 6:**
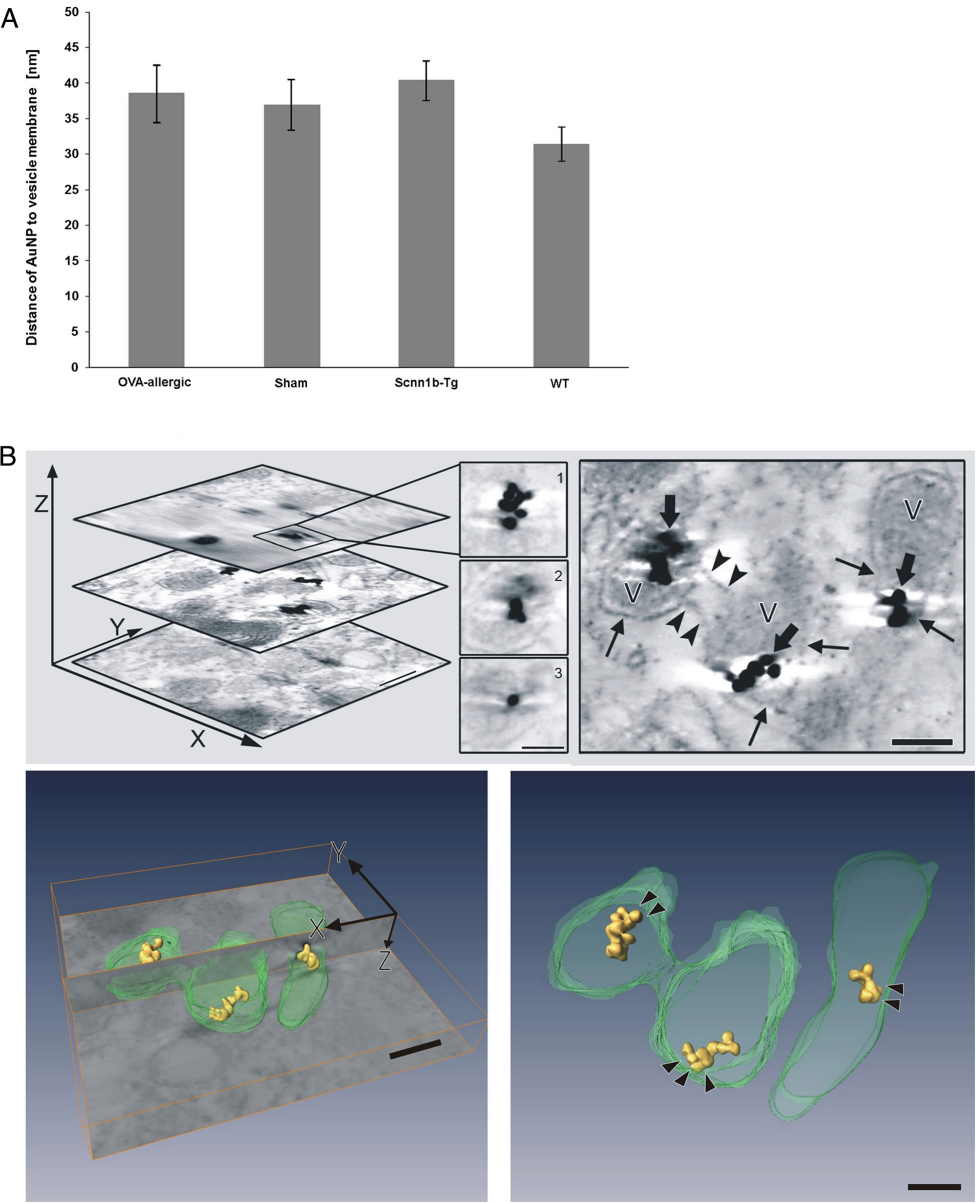
**AuNP localization with respect to the vesicle membrane. (A)** Distance of AuNP to the vesicle membrane as measured on ultrathin sections of BAL macrophages (n = number of measurements). Data are expressed as mean values ± SEM per animal group. **(B)** Electron tomogram of small AuNP clusters in macrophage vesicle. *Upper left*: topmost, middle and lowest image of the 3D stack (bar: 100 nm) and magnified AuNP in different z-heights of the stack (1 - 3, bar: 50 nm). *Upper right*: middle image from the stack indicating connections between two vesicles (arrowheads) and showing AuNP (fat arrows) in close contact to vesicle membrane (arrows). *Lower left*: Bottom image of the tomogram stack (xy-plane) shows the sectioning xz-plane with automatic reconstruction of AuNP (yellow) and manual segmentation of vesicles (green). *Lower right*: reconstruction without the original electron micrograph. Arrowheads point to the space between NP and vesicle membrane. Bars of lower images: 100 nm.

In all groups studied, fungal spores (2.9 μm ± 0.2 μm in diameter, measured on ultrathin sections) were found internalized as single particles. They were localized in macrophage phagosomes of 3.4 μm ± 0.1 μm diameter (Figure 5A), with the vesicle membrane closely surrounding the spores (Figure 4C), resulting in a R_V/P_ = 1.4 ± 0.1. AuNP were mostly found adjacent to the vesicle membrane. Thereby, the distance of AuNP to the membrane tended to be larger in diseased animals of both murine models compared to the healthy mice, though not statistically significant (Figure 6A). The mean distance to the vesicle membrane was 38.5 ± 4.1 nm in BAL cells of OVA-allergic mice vs. 36.9 ± 3.5 nm in sham-sensitized controls and 40.3 ± 2.7 nm in Scnn1b-Tg mice vs. 31.4 ± 2.5 nm in WT littermates. Tomographic reconstructions (n = 3) of single AuNP and small agglomerates, as shown in Figure 6B, confirmed the proximity of AuNP to the vesicle membrane. Rarely, we found AuNP that were not enclosed by a membrane, i.e. localized in the macrophage cytoplasm.In BAL cells from OVA-allergic mice, AuNP were also found in 13.9 ± 8.3% of all observed eosinophils (n = 36), localized in vesicles exceeding NP size and together with other electron dense material of unknown origin (Figure 4D). The few lymphocytes (n = 6) and neutrophils (n = 1) observed in OVA-allergic mice, did not contain any AuNP.

## Discussion

AuNP are of interest for medicine as inhalative drug delivery systems for therapeutic targeting of epithelial cells in pulmonary disease. The action of the drug, however, may be compromised by a previous challenge of the lungs with ambient air particles and/or a rapid clearance of the deposited nanocarriers from the epithelial surface by macrophages. The phagocytic activity of biogenic microparticle challenged airway macrophages with regard to AuNP uptake and lung disease is currently not known. Morphometry combined with electron microscopy allows quantitative assessment of (nano) particle uptake by cells at the individual particle level. Using these methods, we examined the efficiency and the mode of particle internalization in BAL cells after sequential *ex vivo* challenge with a ubiquitous biogenic micrometer-sized particle in ambient air, i.e. fungal spores and an inhalative nanocarrier candidate, i.e. AuNP. To study AuNP uptake in different inflammatory lung disease conditions, we examined BAL cells from OVA-sensitized allergic mice and Scnn1b-Tg COPD mice.

The OVA sensitization model is frequently presented as a mouse model of asthma in the literature, although airway inflammation in this model reflects an acute inflammatory response, rather than the chronic inflammation observed in patients with asthma. This is a general limitation of mouse models of experimental asthma. It can be argued, however, that the type of inflammation obtained in our model mimics key features of airway disease in asthmatic patients, i.e. airway eosinophilia, increased mucin expression and goblet cell metaplasia. In addition, an important point addressed by our acute asthma model in this study is the phagocytosis of nanoparticles during a lung inflammatory response. We were able to show in a chronic asthma model, that the alveolar macrophage population returns to baseline levels after an 8 week cessation of allergen challenge [37]. Since the alveolar macrophage population resumes normal levels in the chronic model, we argue that subsequent inflammation will likely resemble the acute inflammatory response upon successive activation.

Our results show that macrophages are inefficient at taking up AuNP and that lung disease further hampers this process. Macrophages from diseased lungs were structurally altered, larger and in Scnn1b-Tg COPD mice, fewer macrophages internalized AuNP compared to those from healthy controls. These findings are consistent with earlier studies on macrophage clearance of NP of similar size but different materials [7,10,11,18]. In addition, the finding that nanoparticles were taken up by a higher percentage of macrophages from naïve *C3H/HeN:C57BL/6N* mice than from sham-sensitized BALB/c mice points to a strain difference. There are clear differences in the inflammatory response in different mouse strains. In regard to particle uptake, different macrophage phenotypes in the inflammatory models (Th1/Th2) are likely to be an important factor [38]. This further supports the use of the two different models in the present study. The asthma model used displays a Th2 phenotype, while the adult Scnn1b-Tg mice used in this study rather displays a Th1 phenotype.

The larger size of BAL macrophages from diseased mice compared to healthy controls is an indicator of cell activation [39]. Macrophage activation in Scnn1b-Tg mice was supported by other findings including a “foamy” appearance and a higher frequency of substantially enlarged macrophages (up to 36 μm) in these mice, as stated in the results and reported previously [30]. Moreover, in a recent study it was demonstrated that morphological activation of macrophages in Scnn1b-Tg mice is associated with upregulation of macrophage elastase (MMP12) and other signature genes of alternative macrophage activation (*Alox15, Arg1, Chi3l3, Chi3l4, Mgl2, Mmp12, Retnla*) [32]. Macrophages from OVA-allergic mice were even larger than those of Scnn1b-Tg mice. This is in accordance with the inflammation status of the two models, i.e. acute inflammation in the allergic asthmatic and chronic inflammation in COPD mice. By virtue of these findings, one might expect a disease-related increase in phagocytic macrophages. Oppositely, antigen sensitization has been reported to decrease the phagocytic activity of macrophages in experimental asthma in rats [15]. However, both are not the case for AuNP uptake, because the percentage of AuNP-containing macrophages was similar in OVA-allergic mice and sham controls. Less macrophages from Scnn1b-Tg mice contained AuNP compared to those from WT mice. This points to a phenotype change in the macrophage population to a less phagocytic type. This finding is consistent with our recent study on lung-cell distribution of inhaled AuNP, where we found less efficient and delayed NP uptake by surface macrophages in Scnn1b-Tg mice [18]. Decreased uptake of AuNP by macrophages results in their prolonged residence time on the epithelial surface. This facilitates the interaction of AuNP with the epithelial cells or their translocation into tissues beyond. This finding is of interest for therapeutic targeting of epithelial cells in COPD/emphysema using nanocarriers as drug delivery systems. In the case of inhaled harmful NP, this may be an important factor in disease initiation and/or perpetuation including exacerbations.

The intracellular localization of particles within vesicles demonstrates endocytic uptake. Macrophages phagocytosed fungal spores, as expected and consistent with previous findings [26]. Phagocytosis is reflected by the tight enclosure of the microparticle by the vesicle membrane [9]. In our study, vesicles and particles were approximately the same size; hence, the R_V/P_ value was close to 1. Small R_V/P_ values representing particle phagocytosis were also obtained for AuNP clusters consisting of > 10 NP (> 200 nm in diameter). In contrast, single AuNP (20 nm in diameter) were contained in vesicles largely exceeding particle size (R_V/P_ values ~ 20). Such vesicles contained additional other material and were identified as phagosomes, when they contained spores. Thus, it is likely that single AuNP attach to the cell membrane, which is then used by the macrophages to phagocytose larger-sized foreign material, including fungal spores. This “unintentional” uptake of NP correlates with data from previous inhalation studies in rodents using TiO_2_ and Au nanoparticles of approximately 20 nm diameter [11,18]. In the latter study, fast attachment of AuNP to lung cells has been demonstrated [18].

Internalization of AuNP that are attached to the membrane is further supported by the localization of internalized single AuNP and small clusters near and in fairly constant distance to the vesicle membrane. This close particle-membrane association has also been observed in the studies discussed above [11,18]. The distance of 30 – 40 nm between particle and vesicle membrane may indicate binding of AuNP to membrane receptors. This can be visualized in TEM as electron dense material between the particle and the cell membrane. To reach highest possible receptor density in our specimens, we analyzed small AuNP clusters in vesicles and used high resolution electron tomography. However, we did not find any electron dense material between particles and vesicle membrane. Adhesion forces may represent an alternative mechanism for the close localization of AuNP to the vesicle membrane [40].

The following mechanisms are not considered as major pathways of AuNP uptake by airway macrophages. Pinocytosis, because the AuNP-containing vesicles are 4 times larger than what is reported for such endosomes [41,42]. Non-endocytic, passive uptake of particles, found for polystyrene particles of ≤ 200 nm diameter in red blood cells and cytochalasin D treated macrophages [43], because we found AuNP almost exclusively in vesicles. Primary vesicular localization of AuNP in lung cells, i.e. uptake by endocytic mechanisms is in-line with our recent study on lung-cell distribution of inhaled 20-nm AuNP in Scnn1b-Tg and WT mice [18] and with reports from others [44-46].

Our comprehensive statistical analysis of R_V/P_ values in BAL macrophages confirmed for the first time the change in the uptake mechanism determined by particle size. It could not be shown that AuNP uptake by BAL macrophages depends either on the health status of the animals or on the disease model/mouse strain.

Finally, in OVA-allergic mice, we found eosinophils containing AuNP, emphasizing the emerging notion that this cell type may internalize foreign material. Eosinophil uptake of foreign objects has been previously documented with bacteria [47] and antigen-antibody complexes [48]. Uptake of 20-nm AuNP was also demonstrated in peritoneal eosinophils of rats, with NP localized in vesicles of < 100 nm in diameter [49]. Hence, in allergic airway disease, eosinophils may substantially contribute to the clearance of NP from the lung surface. Under these circumstances epithelial targeting with AuNP as nanocarriers for drug delivery may become inefficient, because a substantial amount of NP may be engulfed by additional immune cells present on the airway surface in lung disease and may not reach target cells.

## Conclusions

In summary, our study examined macrophage uptake of AuNP in fungal-spore challenged BAL cells obtained from murine models of allergic asthma and COPD. Macrophages of diseased animals were significantly larger than those of the corresponding controls, indicating a state of activation. Scnn1b-Tg mice had a lower percentage of AuNP containing macrophages, pointing to a change in macrophage phenotype to a less phagocytic population. The resulting prolonged residence time of AuNP on lung surfaces may be favorable for therapeutic targeting of the lung parenchyma by inhaled aerosols. The localization of single AuNP or small particle clusters in large vesicles and close to the membrane, irrespective of the animal model and health status, shows co-uptake of AuNP by macrophages during phagocytic clearance of micrometer-sized particulate deposits from the lung surfaces. The finding that eosinophils in asthmatic lungs significantly internalize AuNP is important for the application of nanocarriers as drug delivery systems for inhalation therapy. The findings of the present study contribute to our understanding of NP uptake by airway macrophages under healthy conditions and in models of the two most relevant inflammatory lung diseases COPD and asthma, and provide a basis for further investigation into the application of medicinal nanoaerosols as well as unraveling the mechanisms responsible for adverse health effects of inhaled NP.

## Abbreviations

AuNP: Gold nanoparticle; BAL: Bronchoalveolar lavage; COPD: Chronic obstructive pulmonary disease; D-MEM: Dulbecco’s Modified Eagle’s Medium; Scnn1b: Epithelial Na^+^ channel β subunit encoded by the *Scnn1b* gene; FELASA: Federation of European Laboratory Animals Science Association; NP: Nanoparticle; OVA: Ovalbumin; PBS: Phosphate buffered saline; SEM: Standard error of the mean; TEM: Transmission electron microscopy; Tg: Transgenic; TiO_2_: Titanium dioxide; UFP: Ultrafine particles; WT: Wild type.

## Competing interests

The authors declare that they have no competing interests.

## Authors’ contributions

MG conceived of the study, designed and supervised it, performed the final data analysis and statistics and wrote the final manuscript. CW performed the particle exposure experiments and part of the microscopic analyses, and contributed to the draft manuscript. SEB contributed to data analysis and to the final manuscript. MC generated and characterized the asthmatic mice, and contributed to the final manuscript. LK contributed to microscopic and data analyses. HG and HR intellectually accompanied the experimental work. MM generated and characterized the Scnn1b-Tg mice, intellectually accompanied the experimental work and contributed to the final manuscript. All authors read and approved the final manuscript.

## Pre-publication history

The pre-publication history for this paper can be accessed here:

http://www.biomedcentral.com/1471-2466/14/116/prepub
